# RETRACTION: KIF20B Promotes Cell Proliferation and May Be a Potential Therapeutic Target in Pancreatic Cancer

**DOI:** 10.1155/jo/9869050

**Published:** 2025-05-06

**Authors:** Journal of Oncology

RETRACTION: J. Chen, C.-C. Zhao, F.-R. Chen, G.-W. Feng, F. Luo, and T. Jiang. “KIF20B Promotes Cell Proliferation and May Be a Potential Therapeutic Target in Pancreatic Cancer,” *Journal of Oncology*, no. 2021 (2021): 1–11. https://doi.org/10.1155/2021/5572402.

The above article, published online on 9 September 2021 in Wiley Online Library (wileyonlinelibrary.com), has been retracted by John Wiley & Sons Ltd.

The retraction has been agreed following an investigation of the concerns raised by *Hoya camphorifolia* on PubPeer [1], which identified multiple instances of inappropriately overlapping figures.

Specifically:

- Supplementary Figure 1b: The “PANC-1 Control” and “BxPC-3 shRNA” panels are duplicates.
- Figure 4(a): The images of the 4 cell culture plates have been duplicated in Figure 3(a) of [2] and Figure 4(a) of [3], while a mirror image has also been identified in Figure 4(a) of [4]. The labels of the cell cultures are different between the 3 publications.
- Figure 5(a): Some tumors (e.g., fourth tumor in the top row) appear to have been rearranged and reused in multiple publications. These include
  • Figure 5(a) of [5] (top row, third tumor)
  • Figure 5(a) of [6] (top row, third tumor)
  • Figure 4(a) of [7] (top row, third tumor)
  • Figure 4(a) of [8] (top row, first tumor)

As a result of the investigation, the data and conclusions of this article are considered unreliable.

The authors disagree with this retraction.
